# Individual- and Community-Level Determinants for Complete Vaccination among Children Aged 12-23 Months in Ethiopia: A Multilevel Analysis

**DOI:** 10.1155/2020/6907395

**Published:** 2020-09-30

**Authors:** Ayal Debie, Getasew Amare, Simegnew Handebo, Mesafint Ewnetu Mekonnen, Getayeneh Antehunegn Tesema

**Affiliations:** ^1^Department of Health Systems and Policy, Institute of Public Health, University of Gondar, Gondar, Ethiopia; ^2^Department of Health Education and Behavioral Sciences, Institute of Public Health, University of Gondar, Gondar, Ethiopia; ^3^Department of Midwifery, School of Health Sciences, College of Medicine and Health Sciences, Bahir Dar University, Bahir Dar, Ethiopia; ^4^Department of Epidemiology and Biostatistics, Institute of Public Health, University of Gondar, Gondar, Ethiopia

## Abstract

**Background:**

Childhood vaccination continues to increase dramatically. In spite of the success of immunization programs to date, millions of children continued to die each year, and sub-Saharan Africa (SSA) accounted for the world's highest neonatal deaths. Childhood vaccination was designed as one of the most effective ways to reduce child mortalities from fatal vaccine-preventable diseases. Therefore, this study is aimed at investigating the individual- and community-level determinants of childhood complete vaccination in Ethiopia.

**Methods:**

A secondary data analysis was done based on the 2016 Ethiopian Demographic and Health Survey (EDHS). A total weighted sample of 1,984 children aged 12-23 months was included for analysis. Considering the hierarchical nature of EDHS data, a two-level multilevel analysis for assessing individual- and community-level determinants of childhood complete vaccination was done. The intraclass correlation coefficient (ICC), Median Odds Ratio (MOR), Proportional Change in Variance (PCV), and deviance (-2LL) were used for model comparison and for checking model fitness. Variables with *p* value < 0.2 in the bivariable multilevel analysis were considered for the multivariable multilevel analysis. In the multivariable multilevel logistic regression analysis, the Adjusted Odds Ratio (AOR) with 95% Confidence Interval (CI) was reported to declare significant determinants of complete childhood vaccination.

**Results:**

Overall complete vaccination status among children aged 12-23 months was 39% (95% CI: 36.8, 41.2). In the multilevel analysis, secondary or above educated mothers (AOR = 2.48; 95% CI: 1.41, 4.36), richest wealth status (AOR = 2.24; 95% CI: 1.16, 4.32), ≥four ANC visits (AOR = 2.77; 95% CI: 1.90-4.02), employed mothers (AOR = 1.66; 95% CI: 1.26, 2.18), urban residence (AOR = 1.84; 95% CI: 1.00, 3.51), and children in city administration (AOR = 2.66; 9% CI: 1.53, 4.62) were positively associated with vaccination status. On the other hand, children with a female household head (AOR = 0.68; 95% CI: 0.48, 0.96) were negatively associated.

**Conclusion:**

Overall, childhood full vaccination status was low compared with the WHO targets. Maternal education, wealth status, ANC visit, maternal occupation, residence, region, and sex of household head were significant predictors of childhood complete vaccination. As a result, it is better to design a compensation mechanism to the costs associated with childhood vaccination for the poor households and strengthen awareness creation for rural residents to improve the access, utilization, and continuum of vaccination service.

## 1. Background

Globally, childhood vaccination continues to increase dramatically [1]. In spite of the overall success of immunization programs to date, about 11 million under-five children continued to die each year [2]. Of these, sub-Saharan Africa (SSA) accounts for 40% of the worlds' under-five mortality [3]. About 27 million children did not receive the full complement of vaccines, and over 2 million people die worldwide per year due to vaccine-preventable diseases [4]. As a result, childhood vaccination was designed as one of the most effective ways to reduce child mortalities [5].

Ethiopia is the country situated in the horn of Africa and has a great geographical diversity. The 2016 EDHS report indicated that vaccination coverage in Ethiopia was low that only 39% of children received all the basic childhood vaccinations [6]. Studies have shown that maternal education, socioeconomic status, antenatal care, delivery, postnatal care, media exposure, perceptions of vaccination, child's place of birth, and region of residence also influence childhood vaccination status [7–11]. Furthermore, the application of multilevel modeling has helped to assess the community/contextual characteristics, such as the region or province of residence [9, 12, 13], community maternal education [12, 13], level of ANC [9], institutional delivery service utilization [12], and community poverty level [9], on childhood vaccination service utilization.

The DHS surveys are designed in such a way that the individual-level characteristics are nested within community-level characteristics. Although several studies had done and documented the determinant factors associated with childhood vaccination in Ethiopia [14–21], the influence of contextual factors on the vaccination status of children had been given less attention. In addition, using a single-level logistic regression analysis technique to analyze data that has a hierarchical structure (i.e., children nested within communities) violates the independence assumptions of regression [22, 23]. Hence, to address these limitations, the multilevel analysis is a highly recommended tool for further analyzing such data.

Therefore, this study is aimed at investigating the individual- and community-level determinants for full vaccination among children aged 12-23 months in Ethiopia based on the 2016 Ethiopian Demographic and Health Survey.

## 2. Method and Materials

### 2.1. Study Settings

**Figure 1:**
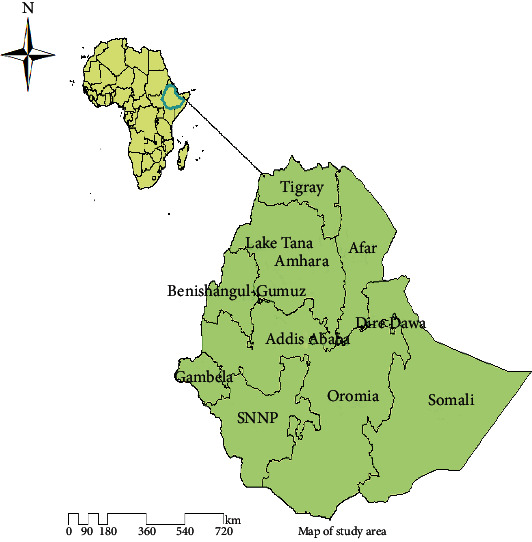
Map of the nine regions and two city administrations of Ethiopia, 2016.

A secondary data analysis was done based on the 2016 Ethiopian Demographic and Health Survey (EDHS). The 2016 EDHS is the fourth DHS survey in Ethiopia collected in the nine administrative regions, namely, Tigray, Afar, Amhara, Benishangul-Gumuz, Gambela, Harari, Oromia, Somali, and Southern Nations, Nationalities, and People's Region (SNNP), and two city administrative regions (Addis Ababa and Dire Dawa) (Figure 1). Most of the population of Ethiopia is an agrarian society, and about 43 percent of the Gross Domestic Product (GDP) of the country has been accounted for by agriculture, and 84% of the population lives in rural areas. More than 80 percent of the country's total population lives in the regional states of Amhara, Oromia, and SNNP [24]. In addition, Ethiopia is the thirteenth in the world and the second in Africa's most populous countries with a 4.46 fertility rate. Ethiopia has followed 3 tiers of preventive healthcare system approaches. These are primary-level healthcare comprising of a primary hospital, health center, and health post; secondary-level healthcare (general hospital); and tertiary-level healthcare (specialized hospital). The number of hospitals varies from region to region in relation to the size of the population. The Oromia region has the highest number of hospitals (30), and only one hospital is found in the Gambela region [25].

### 2.2. Sample and Population

All children aged 12-23 months in Ethiopia were the source population, while children aged 12-23 months in the selected enumeration areas before five years of the survey were the study population. In EDHS, a stratified two-stage cluster sampling technique was employed to select the study participants using the 2007 Population and Housing Census (PHC) as a sampling frame. Stratification was achieved by separating each region into urban and rural areas. A total of 21 sampling strata have been created because the Addis Ababa region is entirely urban. In the first stage, 645 enumeration areas (EAs) were selected proportionally to the size of EAs with independent selection from the sampling stratum. Consecutively, a complete list of the households was carried out in all selected EAs before the actual data collection period, and 28 households were selected using a systematic sampling technique. Of these, 18,008 households and 16,583 eligible women were included, and the detailed sampling procedure was presented in the full 2016 EDHS report [6].

### 2.3. Measurements of Variables

The full vaccination status of children in Ethiopia was the outcome variable for this study. The EDHS data about vaccination were collected from verbal reports of the mother and data extraction from the childhood immunization card. The detailed report is found in the 2016 EDHS report. A child that received one dose of BCG, 3 doses of pentavalent, 3 doses of polio, two doses of rota, three doses of PCV, and one dose of measles was considered fully vaccinated and categorized as “yes,” and the remaining was categorized as “no.” The response variable for the *i*^th^ child is represented by a random variable *Y*_*i*_ with two possible values coded as 1 and 0. As a result, the response variable of the *i*^th^ child *Y*_*i*_ was measured as a dichotomous variable with possible values of “*Y*_*i*_ = 1” if the *i*^th^ child was fully vaccinated and “*Y*_*i*_ = 0” if the child was not fully vaccinated. Accordingly, consistent with the objective of the study and the hierarchical structural nature of the EDHS data, the women were nested within the cluster/community. As a result, we considered two levels of the independent variables. These are the individual-level (level one) factors including individual sociodemographic and economic factors, such as age, maternal education, paternal education, media exposure, wealth index, maternal occupation, health insurance, and sex of head of the household, and maternal obstetric-related factors, such as ANC visit, parity, preceding birth interval, and birth order. On the other hand, the community-level (level two) factors include the characteristics of the community such as the region, residence, community media exposure, community women education, region, and place of residence. This might help us to see whether the cluster-level variables had an effect on full childhood vaccination status. Community-level variables had two sources. These are direct community- and aggregated community-level variables used in the analysis of community-level variables. Some of the community-level variables such as community education and media exposure were an aggregate result of the individual data and categorized as low or high using the median value since the EDH data were not normally distributed.

### 2.4. Data Management and Analyses

The data were weighted using sampling weight, primary sampling unit, and strata before any statistical analyses to restore the representativeness of the survey and to get reliable statistical estimates. Cross-tabulations and summary statistics were conducted to describe the study population using STATA version 14 software. In the EDHS data, children are nested within a cluster, and children within the same cluster might be more similar to each other than children in the rest of the country. This violates the assumption of a simple binary logistic regression model such as the independence of observations and equal variance across clusters. Therefore, a multilevel logistic regression model (both fixed and random effect) was fitted to take into account the clustering effect. Model comparison was done based on deviance since the models were nested. The likelihood ratio, intraclass correlation coefficient (ICC), Median Odds Ratio (MOR), and Proportional Change in Variance (PCV) were computed to measure the variation between clusters. The intraclass correlation coefficient (ICC) quantifies the degree of heterogeneity of childhood full vaccination between the clusters, or it is the proportion of the total observed individual variation in childhood full vaccination attributed to between-cluster variations.

ICC = *б*2/(*б*2 + *π*2/3) [26], but MOR is quantifying the variation or heterogeneity in outcomes between clusters and defined as the median value of the odds ratio between the cluster at high likelihood of full vaccination and cluster at lower likelihood when randomly picking out two clusters or EAs. $\text{MOR}=\exp\;\left(\sqrt{2*\partial 2*0.6745}\right)\sim \text{MOR}=\exp\;\left(0.95*\partial \right)$ [27]. *∂*^2^ indicates the cluster variance, and PCV measures the total variation attributed to individual- and community-level factors in the multilevel model as compared to the null model:

(1) $$\begin{array}{c} \text{PCV}=\frac{\mathrm{var}\left(\text{null model}\right)-\mathrm{var}\;\left(\text{full model}\right)}{\mathrm{var}\left(\text{null model}\right)}. \end{array}$$

A two-level, at individual and community (cluster) levels, multilevel multivariable logistic regression was used to analyze factors associated with childhood full vaccination. Furthermore, four models were constructed for the multilevel logistic regression analysis. The first model was an empty model without any explanatory variable/s to determine the extent of cluster variation on full vaccination. The second model was adjusted with individual-level variables; the third model was adjusted for community-level variables, while the fourth was fitted with both individual- and community-level variables simultaneously. As a result, a model with the lowest deviance was chosen. Multicollinearity was also checked using the Variance Inflation Factor (VIF), and VIF < 10 and tolerance greater than 0.1 were used to declare the absence of multicollinearity. Variables with *p* values < 0.2 in the bivariable analysis for both individual- and community-level factors were fitted in the multivariable model. Adjusted Odds Ratio (AOR) with a 95% Confidence Interval (CI) and *p* values < 0.05 in the multivariable model were used to declare a significant association with childhood full vaccination.

## 3. Results

### 3.1. Individual-Level Sociodemographic and Economic Characteristics

A total of 1,984 children aged 12-23 months were included in the analysis. Of these, 1,046 (52.7%) of the children were born to mothers aged 25-34 years and 1,250 (63.0%) were born to mothers with no formal education. About one-fourth of the children (25.4%) were from the poorest household wealth status and 284 (14.3%) were from the richest household wealth index (Table 1).

### 3.2. Community-Level Sociodemographic Characteristics

More than ninety percent (90.8%) of the children were from developed regions of Ethiopia, namely, Amhara, Oromia, SNNP, and Tigray regions, while 6.2 and 3.3% of children were from emerging regions and city administrations of Ethiopia, respectively (Table 2).

### 3.3. Childhood Full Vaccination Status

**Figure 2:**
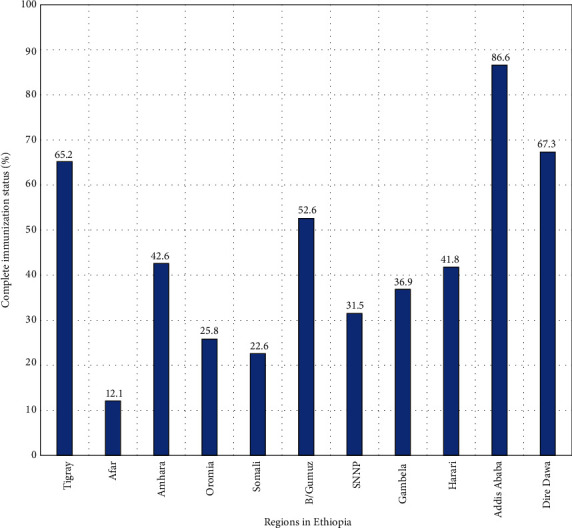
Childhood complete vaccination coverage across regions in Ethiopia, EDHS 2016.

The overall childhood full vaccination coverage among children aged 12-23 months in Ethiopia was 39% (95% CI: 36.8, 41.2). The vaccination status of children was significantly varied across regions of the country. Subsequently, the lowest vaccination coverage was observed in the Afar region (12.1%), while the highest was seen in Addis Ababa (86.6%) (Figure 2).

### 3.4. Individual- and Community-Level Determinants for Childhood Full Vaccination

#### 3.4.1. Random Effects Analysis

The ICC in the null model was 50%, which revealed that about 50% of the total variability of childhood full vaccination was due to the differences between clusters, while the remaining unexplained 50% was attributable to the individual differences. Moreover, the MOR was 5.60 in the null model, and this indicated that there was a variation between clusters. A child at the cluster with high likelihood of being fully vaccinated had 5.6 times higher odds to be fully vaccinated compared with a child at a cluster with low likelihood of fully vaccinated during random selection of children at two different clusters. Fifty-six percent of the variability in childhood full vaccination was explained by the full model, and deviance was used for model comparison. As a result, the final model was the best-fitted model since it had the lowest deviance (Table 3).

#### 3.4.2. Fixed Effects Analysis

For the multivariable multilevel logistic regression analysis, maternal education, wealth status, maternal occupation status, ANC visit, residence, region, and sex of household head were significant predictors of full childhood immunization. Secondary or above educated mothers had 2.48 (AOR = 2.48; 95% CI: 1.41, 4.36) times higher odds of fully vaccinating their children than mothers with no formal education. Children in the middle, richer, and richest household's wealth status had 1.68 (AOR = 1.68; 95% CI: 1.10, 2.59), 2.10 (AOR = 2.10; 95% CI: 1.33, 3.32), and 2.24 (AOR = 2.24; 95% CI: 1.16, 4.32) times higher odds of full vaccination than children in the poorest households, respectively. Mothers who attended two, three, and four or above ANC visits during pregnancy were 2.60 (AOR = 2.60; 95% CI: 1.53, 4.40), 3.77 (AOR = 3.77; 95% CI: 2.48, 5.75), and 2.77 (AOR = 2.77; 95% CI: 1.90-4.02) times higher odds of fully vaccinating their children compared to mothers with no ANC visit, respectively. Employed mothers had 1.66 (AOR = 1.66; 95% CI: 1.26, 2.18) times higher odds of fully vaccinating their children compared with unemployed mothers. Urban resident children had an 84% (AOR = 1.84; 95% CI: 1.00, 3.51) increased odds of childhood full vaccination compared with the rural residents. Children from city administration regions in the country were 2.66 (AOR = 2.66; 9% CI: 1.53, 4.62) times more likely to be fully vaccinated compared to children from emerging regions. In addition, households with a female household head had 32% (AOR = 0.68; 95% CI: 0.48, 0.96) decreased odds of childhood full vaccination status (Table 4).

## 4. Discussion

Childhood full vaccination coverage among children aged 12-23 months in Ethiopia was 39% (95% CI: 36.8, 41.2) that significantly varied across regions. It could be due to disparity in healthcare service availability and utilization, such as family planning, ANC, and other reproductive health services within the country, and this could have an impact on the vaccination service utilization of women for their children [28–30].

Maternal education, maternal occupation, ANC visit, sex of household head, wealth status, region, and residence were found to be significant determinant factors of full childhood immunization. In this study, maternal education was positively associated with childhood full vaccination. Children born to mothers who attained secondary education or above were more likely to be fully vaccinated compared to children born to mothers with no formal education. It was consistent with previous study findings reported in Ethiopia [9] and India [31].

This might be due to the fact that educated mothers might have better awareness towards the advantage of childhood vaccination and about vaccine-preventable diseases; this could increase their chance of childhood vaccination service utilization. The other possible justification might be mothers with formal education might have better access to media exposure, such as radio, television, and newspapers.

The other significant predictors of childhood full immunization were household wealth status. The study showed that children with rich household wealth status were more likely to be fully vaccinated than children with poor household wealth status. This finding was in line with the studies conducted in Ethiopia [8, 15] and Bangladesh [32]. The possible justification might be due to the differences in the healthcare accesses between poor and rich households. As a result, children with the poorest household wealth status were marginalized and difficult to get the healthcare services since the poor households might spend high costs and their times to maintain their daily lives.

Mothers having an ANC follow-up during pregnancy are more likely to fully vaccinate their children compared with mothers with no ANC follow-up. This finding was consistent with the studies done in Ethiopia [9, 15], India [33], Senegal [10], and Bangladesh [32]. The possible justification might be women who had ANC follow-up during pregnancy might get counseling services about vaccination including the immunization schedule since ANC is one of the means to create awareness about childhood vaccination. Accordingly, children with employed mothers were associated with an increased childhood full vaccination compared with unemployed mothers. This finding was in line with a study done in Nigeria [34]. This might be due to the fact that employed mothers had better information access since they might have better access to media and about disease prevention, such as vaccination and vaccine-preventable diseases.

Another significant variable of childhood full vaccination was the place of residence. As a result, children with urban residence were significantly associated with childhood full vaccination than rural residents. This was consistent with studies done in Ethiopia [9], Philippines [35], and Nigeria [36]. This could be due to the disparities in the distribution of healthcare facilities in Ethiopia, mainly concentrated in urban areas. They might have better access to healthcare services, media exposure, childhood vaccination, vaccine-preventable diseases, and education.

Another important predictor variable was the female household head; the likelihood of childhood full vaccination was lower in households with female household heads compared with male-headed households. The finding was in line with a study done in Togo [37]. The possible justification might be due to the reasons that households with a female head might predispose mothers to additional high workload including family responsibilities; perhaps, mothers could not have adequate time for childhood vaccination.

This study has strengths and limitations. This study was based on the nationally representative EDHS survey in Ethiopia that was weighted, and multilevel analysis was done to get a reliable estimate and standard error. Besides, this study was based on a large sample size that had adequate power to detect the true effect of the independent variables. As a limitation, since the study used cross-sectional data, we are unable to establish a causal relationship between full childhood vaccination and the identified independent variables. Secondly, information about basic vaccinations was collected by reviewing children who had vaccination cards and mothers' verbal responses since all children did not have the vaccination card which was found to be prone to recall bias. Besides, variables such as mother's knowledge and attitude towards vaccination and perceived quality of services were not found in EDHS.

## 5. Conclusion

The overall childhood full vaccination status was low compared with the World Health Organization targets. Maternal education, household wealth status, ANC visit, employment status of mothers, residence of children, and household head were significantly associated with childhood full vaccination. As a result, enhancing maternal education and ANC service utilization can help to improve childhood vaccination. Similarly, it is better to design a compensation mechanism to the costs associated with childhood vaccination for the poor households and strengthen awareness creation for rural residents to improve the access, utilization, and continuum of vaccination service.

## Figures and Tables

**Table 1:** Individual-level characteristics of mothers and their children in Ethiopia, EDHS 2016.

Variables	Categories	Frequency (*n* = 1,984)	Percent (%)
Religion	Orthodox	690	34.8
	Muslim	779	39.3
	Protestant	439	22.1
	Others	76	3.8
Maternal age in years	<25	489	24.7
	25-34	1,046	52.7
	≥35	449	22.6
Maternal education	No education	1,250	63.0
	Primary	568	28.6
	Secondary or above	166	8.4
Husband education	No education	1,006	50.7
	Primary	737	37.2
	Secondary	145	7.3
	Higher	96	4.8
Wealth status	Poorest	503	25.4
	Poorer	390	19.6
	Middle	447	22.5
	Rich	360	18.2
	Richest	284	14.3
Marital status	Unmarried	123	6.2
	Married	1,861	93.8
Media exposure	No	7,375	66.9
	Yes	3,647	33.1
Employment/occupation	Yes	897	45.2
	No	1,087	54.8
Sex of household head	Male	1,694	85.4
	Female	290	14.6
Place of delivery	Home	1,232	62.1
	Health facility	752	37.9
PNC service utilization	No	1,834	92.4
	Yes	150	7.6
Use of ANC services	No	705	35.5
	Yes	1,279	64.5
Covered by health insurance	No	1,905	96.0
	Yes	79	4.0
Birth order	1-3	965	48.6
	4-6	646	32.6
	7^+^	373	18.8
Parity	1	337	17.0
	2-5	1,079	54.4
	6^+^	568	28.6
Child size at birth	Large	671	33.8
	Average	765	38.6
	Small	548	27.6

**Table 2:** Community-level characteristics of mothers and their children in Ethiopia, 2016 (*n* = 1,984).

Variables	Categories	Frequency	Percent (%)
Regions	Developed	1,799	90.8
	Emerging	119	6.2
	City administrations	64	3.3
Residence	Urban	228	11.5
	Rural	1,756	88.5
Community women education	Lowest	1,137	57.3
	Highest	847	42.7
Community media exposure	Lowest	1,199	60.4
	Highest	785	39.6

**Table 3:** Random effects analysis for childhood full immunization in Ethiopia based on EDHS 2016.

Parameter	Null model	Model 1	Model 2	Model 3
Community-level variance (SE)	3.29 (0.52)	1.38 (0.28)	2.03 (0.36)	1.44 (0.29)
Log likelihood	-1,153.72	-1,005.60	-1,059.99	-995.0
Deviance	2,307.44	2,011.20	2,119.98	1,990.0
MOR	5.60 (4.37, 7.49)	3.05 (2.49, 3.90)	3.87 (3.13, 5.00)	3.13 (2.55, 4.02)
PCV	Ref	0.46	0.38	0.56
ICC	0.50	0.30	0.38	0.30

**Table 4:** Multivariable multilevel logistic regression analysis of individual- and community-level determinants of childhood full immunization in Ethiopia, EDHS 2016.

Variables	Null model	Model 1	Model 2	Model 3
*Individual-level factors*				
Mode of delivery				
Vaginal delivery	—	1		1
Cesarean delivery	—	1.47 (0.70, 3.09)	—	1.17 (0.55, 2.51)
Birth order				
1-3	—	1		1
4-6	—	1.06 (0.78, 1.45)	—	1.08 (0.79, 1.48)
7^+^	—	0.83 (0.56, 1.23)	—	0.86 (0.58, 1.28)
Place of delivery				
Home	—	1		1
Health facility	—	1.51 (1.10, 2.06)^∗^	—	1.37 (0.99, 1.88)
H/insurance coverage				
No	—	1		1
Yes	—	1.40 (0.69, 2.84)	—	1.36 (0.66, 2.79)
Maternal education				
No education	—	1		1
Primary	—	1.43 (1.04, 1.99)^∗^	—	1.36 (0.96, 1.92)
Secondary or higher	—	2.82 (1.64, 4.85)^∗∗^	—	2.48 (1.41, 4.36)^∗∗^
Husband education				
No education	—	1		1
Primary	—	1.11 (0.82, 1.51)	—	1.08 (0.79, 1.47)
Secondary	—	0.97 (0.61, 1.57)	—	0.99 (0.61, 1.61)
Higher	—	0.61 (0.34, 1.12)	—	0.66 (0.36, 1.22)
No. of ANC visit				
No	—	1		1
1	—	1.68 (0.87, 3.26)	—	1.59 (0.82, 3.10)
2	—	2.71 (1.61, 4.56)^∗∗^	—	2.60 (1.53, 4.40)^∗∗^
3	—	3.89 (1.51, 3.70)^∗∗^	—	3.77 (2.48, 5.75)^∗∗^
4^+^	—	2.96 (2.04, 4.28)^∗∗^	—	2.77 (1.90, 4.02)^∗∗^
Wealth status				
Poorest	—	1		1
Poorer	—	2.10 (1.41, 3.13)^∗∗^	—	1.90 (1.27, 2.85)^∗^
Middle	—	1.87 (1.24, 2.85)^∗∗^	—	1.68 (1.10, 2.59)^∗∗^
Richer	—	2.37 (1.51, 3.70)^∗∗^	—	2.10 (1.33, 3.32)^∗∗^
Richest	—	4.82 (2.92, 7.98)^∗∗^	—	2.24 (1.16, 4.32)^∗∗^
Sex of H/head				
Female	—	0.65 (0.47, 0.92)^∗^	—	0.68 (0.48, 0.96)^∗∗^
Male	—	1		1
Media exposure				
No	—	1		1
Yes	—	1.18 (0.86, 1.62)	—	1.09 (0.78, 1.52)
Women employment				
Unemployed	—	1		1
Employed	—	1.63 (1.25, 2.13)^∗∗^	—	1.66 (1.26, 2.18)^∗∗^
PNC utilization				
No	—	1		1
Yes	—	1.01 (0.65, 1.57)	—	1.09 (0.67, 1.70)
*Community-level factors*				
Community women education				
Lower	—		1	1
Higher	—	—	2.25 (1.53, 3.33)^∗∗^	1.25 (0.84, 1.86)
Residence				
Rural	—		1	1
Urban	—	—	3.39 (1.99, 5.79)^∗∗^	1.84 (1.00, 3.51)^∗^
Community media				
Lower	—		1	1
Higher	—	—	1.76 (1.16, 2.67)^∗∗^	1.13 (0.75, 1.72)
Regions				
Emerging regions	—		1	1
Developed regions	—	—	2.40 (1.58, 3.64)^∗∗^	1.45 (0.96, 2.19)
City administration	—	—	4.31 (2.43, 7.65)^∗∗^	2.66 (1.53, 4.62)^∗∗^
Constant	0.49 (0.40, 0.60)	0.06 (0.04, 0.10)	0.113 (0.08, 0.17)	0.05 (0.03, 0.08)

## Data Availability

The datasets used during the current study are available at the MEASURE DHS website: http://www.measuredhs.com.

## Abbreviations

ANC: Antenatal care
DPT: Diphtheria, pertussis, and tetanus
EA: Enumeration area
EDHS: Ethiopia Demographic and Health Survey
EPI: Expanded program of immunization
SD: Standard deviation
USD: US dollar
VPD: Vaccine-preventable diseases
WHO: World Health Organization.

## Additional Points

The 2016 EDHS data is available to the general public by request in different formats from the MEASURE DHS website http://www.measuredhs.com. We submitted a request to the MEASURE DHS website by briefly stating the objectives of this analysis and thereafter received permission to download the maternal and children's dataset in STATA format.
